# Effects of PMTO in Foster Families with Children with Behavior Problems: A Randomized Controlled Trial

**DOI:** 10.1007/s10826-016-0579-2

**Published:** 2016-10-12

**Authors:** Anne M. Maaskant, Floor B. van Rooij, Geertjan J. Overbeek, Frans J. Oort, Maureen Arntz, Jo M. A. Hermanns

**Affiliations:** 1 Research Institute Child Development and Education, University of Amsterdam, Amsterdam, The Netherlands; 2 H&S Consult, Woerden, The Netherlands

**Keywords:** Foster care, Parent Management Training Oregon, Child behavior problems, Parenting stress, Randomized controlled trial

## Abstract

The present randomized controlled trial examined the effectiveness of Parent Management Training Oregon for foster parents with foster children (aged 4–12) with severe externalizing behavior problems in long-term foster care arrangements. Foster children’s behavior problems are challenging for foster parents and increase the risk of placement breakdown. There is little evidence for the effectiveness of established interventions to improve child and parent functioning in foster families. The goal of Parent Management Training Oregon, a relatively long and intensive (6–9 months, with weekly sessions) parent management training, is to reduce children’s problem behavior through improvement of parenting practices. We specifically investigated whether Parent Management Training Oregon is effective to reduce foster parenting stress. A significant effect of Parent Management Training Oregon, compared to Care as Usual was expected on reduced parenting stress improved parenting practices, and on reduced child behavior problems. Multi-informant (foster mothers, foster fathers, and teachers) data were used from 86 foster families (46 Parent Management Training Oregon, 40 Care as Usual) using a pre-posttest design. Multilevel analyses based on the intention to treat principle (retention rate 73 %) showed that Parent Management Training Oregon, compared to Care as Usual, reduced general levels of parenting stress as well as child related stress and parent-related stress (small to medium effect sizes). The clinical significance of this effect was, however, limited. Compared to a decrease in the Care as Usual group, Parent Management Training Oregon helped foster mothers to maintain parental warmth (small effect size). There were no other effects of Parent Management Training Oregon on self-reported parenting behaviors. Child behavior problems were reduced in both conditions, indicating no additive effects of Parent Management Training Oregon to Care as Usual on child functioning. The potential implication of reduced foster parenting stress for placement stability is discussed.

## Introduction

Children in foster care have substantially higher levels of behavioral and emotional problems than children in the general population (Burns et al. 2004; Landsverk et al. 2006; Maaskant et al. 2014). These problems increase parental stress of foster parents (Vanderfaeillie et al. 2012) and often lead to placement disruption (for a meta-analysis see Oosterman et al. 2007; Van Rooij et al. 2015). Placement disruption in turn increases the risk of consecutive unstable placements for foster children (e.g., Farmer 1996; Rubin et al. 2007). Moreover, behavioral and emotional problems and unstable placements negatively affect children’s developmental outcomes, including increased risk for internalizing and externalizing behavior problems in later life, drug use, school dropout, and delinquency (Aarons et al. 2010; Herrenkohl et al. 2003; Newton et al. 2000).

Given this high risk prognosis for children in foster care, it is pivotal to stimulate sensitive and consistent parenting in foster parents. If foster parents are supported in a way they feel less stressed and more competent to handle disruptive behaviors of their foster child, effective parenting is supposed to increase (Vanderfaeillie et al. 2012) and child behavior is supposed to improve (Chamberlain et al. 2008; Deković et al. 2010). As a consequence, the risk for untimely placement breakdown is supposed to reduce (Chamberlain et al. 2008).

The past decennium several review studies and meta-analyses have reviewed the conducted studies on the effectiveness of parenting interventions in foster care (e.g., Dorsey et al. 2008; Kinsey and Schlosser 2013; Leve et al. 2012; Macdonald and Turner 2008; Rork and McNeil 2011; Turner et al. 2009). These reviews show an extensive heterogeneity in outcome measures among the individual studies. Few studies focused on the effectiveness of parent training on parental stress of foster parents. The three studies that did, demonstrated small to medium effects of foster parenting interventions on parenting stress (Multidimensional Treatment Foster Care for Preschoolers: Fisher and Stoolmiller 2008; Incredible Years (IY): Bywater et al. 2010; Parent-Child Interaction Therapy: Timmer et al. 2006). More studies focused on foster child behavior and foster parenting behavior. Concerning foster child behavior and foster parenting behavior, the reviews makes clear that overall, widely used curricula of foster parenting interventions have limited impact (e.g., Model Approach to Partnership in Parenting, Parent Resources for Information, Development and Education; Dorsey et al. 2008; Turner et al. 2009). Interventions may have been too “light” for foster parents, relatively brief and not adjusted to meet the individual needs of foster parents (Turner et al. 2009). Individualized and intensive skill-based training for foster parents, *after* a child is placed in their home, seems to be an important requirement for positive change (Dorsey et al. 2008; Kinsey and Schlosser 2013; Leve et al. 2012).

Besides investigating overall intervention effects, it is crucial to determine for whom interventions work best (Lundahl et al. 2006). In foster care samples, there is a dearth of research on moderators of the effectiveness of parenting interventions. However, one study did show that the initial level of child behavior problems moderated the effects of the KEEP program on parenting behavior, such that the foster parents of children with more disruptive behaviors benefited more (Chamberlain et al. 2008). In general populations, children with high levels of initial problem behavior also tend to show better outcomes from parenting interventions (Gardner et al. 2015; Hautmann et al. 2010; Leijten et al. 2013; Lundahl et al. 2006). Nevertheless, some other studies found contrary or no results (see review by McMahon et al. 2006). Next, younger children tend to benefit more from parenting interventions than older children (Lundahl et al. 2006; McCart et al. 2006) and boys seem to benefit more than girls from parenting interventions in terms of reduced behavior problems (Gardner et al. 2010). Such gender effects are, however, also inconsistent across some studies (McMahon et al. 2006). Furthermore, families with depressed parents seem to benefit more from parenting interventions in terms of improvement of problem behavior of their children and parenting skills (Gardner et al. 2015; Beauchaine et al. 2005; Sigmarsdόttir et al. 2013). The role of child age, gender and parental depression on the effectiveness of parenting interventions in foster families may be the same as in the general population, but has never been investigated.

In sum, previous research shows little evidence for the effectiveness of current parenting interventions aimed at foster parents for reducing child behavior problems and improving effective parenting behaviors. Furthermore, despite the widespread knowledge that rearing foster children can have a negative impact on the psychological functioning of foster parents, the extent to which interventions can help to diminish parental stress and for whom has been largely ignored. Elaborating on the conclusions of Turner et al. (2009) that the current interventions may be too light, too short and insufficiently individualized, there is a strong need for research on promising interventions for improving child and parent functioning in foster families. Parent Management Training Oregon (PMTO) is an internationally well-established parenting intervention that has recently been implemented in Dutch traditional long-term foster care. To note, in the Netherlands, in contrast to short-term foster care, aimed at treating a child or parent for the purpose of returning the child to his or her birth-family, long-term foster care is provided till the child reaches adulthood, centering on the continuity and the child’s right to a stable rearing situation. Dutch foster children are rarely adopted.

PMTO is an intensive (mostly 6–9 months with weekly sessions), individual parenting intervention in which intervention goals are set in agreement between trainer and parents. PMTO treatment is based on the social interaction learning model (SIL), which combines the principles of social learning, social interaction and behavioral perspectives (Forgatch et al. 2004; Reid et al. 2002). SIL emphasizes the importance of the social context in the development of children (Patterson 2005). Contextual factors (e.g., family structure transitions, parents’ stress-level and children’s temperament) are expected to have indirect effects on child outcomes, and are mediated by coercive processes and ineffective parenting skills (Forgatch et al. 2005b). Coercive cycles in family interactions are initiated when children and parents reinforce each other’s negative behavior, and these cycles often flourish in stressful contexts (Forgatch et al. 2005b; Patterson et al. 1982). In relationships characterized by coercive interactions, parental expression of warmth and encouragement tend to be scarce, and the children are rarely reinforced for developing positive skills (Hagen et al. 2011). Once coercive processes are established, they tend to be maintained by both the parent and child. The main focus of PMTO is enhancing effective and positive parenting practices, and diminishing coercive practices while making relevant adaptations for high risk contextual factors (e.g., divorce; Forgatch et al. 2005a). The five central parenting skills are: limit setting and discipline, monitoring and supervision, problem solving, positive involvement, and skill encouragement (Patterson 2005). In addition to the core parenting practices, PMTO incorporates the supporting parenting components of identifying and regulating emotions, enhancing communication, giving clear directions, and tracking behavior.

PMTO was effective in improving effective parenting skills and child behavior in several international clinical and prevention samples, and for a broad range of families (traditional families, stepfamilies, single parents, and ethnic minorities; Bullard et al. 2010; DeGarmo and Forgatch 2005; Forgatch et al. 2005a; Martinez and Forgatch 2001; Ogden and Hagen 2008; Patterson et al. 1982). A Blueprints meta-analysis of three PMTO studies found small but consistent positive effects of the program on multiple child-outcomes (Blueprints for Healthy Youth Development 2016; mean effect size 0.20). Regarding parenting practice outcomes, effect sizes were on average 0.33 (DeGarmo et al. 2004; DeGarmo and Forgatch 2007; Ogden and Hagen 2008), although one Randomized Controlled Trial (RCT) in Iceland found no effects of PMTO on maternal parenting practices (Sigmarsdόttir et al. 2013). Until now, the effectiveness of PMTO in real-life foster care (i.e., a routine foster care setting), and its effects on parenting stress have not yet been investigated.

The present study is an effectiveness study in a Dutch ‘real world’ long-term foster care setting, with a high risk foster care sample, that tested the added value of PMTO compared to care as usual. We employed an RCT design to examine the effects of PMTO on parenting stress (related to parent self and to the child), parenting behavior (warmth, responsiveness, explaining, autonomy granting, strictness, and discipline) and children’s problem behavior (externalizing and internalizing problems). We hypothesized that PMTO, compared to care as usual (CAU), resulted in (1) reduced parenting stress, (2) improved parenting behavior, and (3) reduced child behavior problems, as reported by foster mothers and foster fathers (and as nested in one family). Next, we explored for whom PMTO worked best. We expected larger effects of PMTO for foster parents of younger children, boys, children with higher initial severity of behavior problems, and for parents with higher initial levels of depressed feelings.

## Method

### Participants

This study aimed to acquire a sample of 75 foster children with elevated behavioral problems (assuming effect size = 0.33; autocorrelation = 0.5, and *α* = .05, a sample of 75 suffices to achieve power = 0.80).

**Fig. 1:**
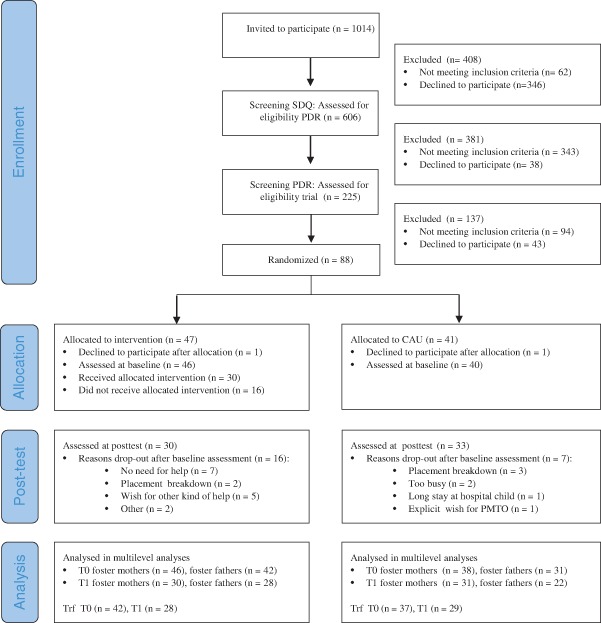
Flow chart

Foster families (among ongoing placements) were recruited using a two-stage screening procedure in collaboration with three regional foster care institutions where PMTO delivery was already implemented (Fig. 1). Families were gradually enrolled between January 2011 and April 2014. If a family that was not yet invited to participate had an urgent need for help (as indicated by the supervisor) the family was immediately invited for the screening procedure, but only if the foster parents and supervisor agreed following the regular screening and randomization procedures carried out by the independent research team.

In a first stage, all foster parents of children aged between 4-years-old and 11-years-old placed in foster care for at least 1 year, were invited to fill in the Strengths and Difficulties Questionnaire (SDQ, Van Widenfelt et al. 2003). In total, the foster parents of 1014 foster children received the SDQ, and 668 responded (66 %). The foster parents of 263 children had a Total Difficulties Score above the clinical cut off score of 14 (see Dutch Manual of the SDQ 2006) and were approached to participate in the second stage of the screening procedure. In case multiple foster children in a foster family scored 14 or higher, the second screening stage focused on the foster child with the highest score.

The second stage involved a telephone interview of five minutes on three consecutive days using the Parent Daily Report (PDR, Chamberlain et al. 2006), a measurement used for screening of daily behavior problems (e.g., hit by the child, lying of child, tantrums). The aim of this interview was to target a sample with foster families who daily experience severe levels (a mean number of more than five different types of problem behavior each day) of child behavior problems. In total, the foster parents of 225 foster children agreed to participate (86 %). Within this group, the foster parents with a PDR score >5 were considered at a high risk for placement breakdown (Chamberlain et al. 2006; Hurlburt et al. 2010) and eligible to participate in the RCT. Families that already received PMTO were excluded. The foster parents of 131 children were eligible to participate in the RCT and of this group 88 (67 %) agreed to take part in the study. Two families dropped out directly after randomization (without baseline assessment), leaving a sample with one or both foster parents of 86 foster children (66 % of all eligible families). With a mean time of 10 months from baseline, the foster parents of 63 foster children (30 of the PMTO condition, 33 of the CAU condition) completed posttest assessments (see flowchart for reasons drop out).

### Procedure

#### Design

This study reports on baseline and post-intervention data collected in a RCT in the Netherlands. The study received ethical approval from the Ethical Committee of the Research Institute of Child Development and Education of the University of Amsterdam and was registered at the Dutch Trial Register (NTR4282).

#### Randomization

Eligible participants were randomized following a 1:1 allocation ratio to either the intervention group (*n* = 46) or control group (*n* = 40) (see Fig. 1, flowchart). Randomization was undertaken by coin tossing by the second author, who was not involved in the enrollment process and blind for personal information of eligible participants. All staff and counselors of foster care organizations were blind for the randomization process. The researchers were not involved with the implementation and execution of PMTO.

#### PMTO

The PMTO program is fully manualized (Forgatch 1994). The central role of the PMTO therapist is to teach and coach parents by role play, and modeling exercises in the use of effective parenting strategies. Nevertheless, the central parenting skills and supporting parenting components offered by the therapists depend on the specific goals set for each family. Internationally the mean number of individual treatment sessions is about 25 (depending on the set goals) and sessions are generally once a week (Factsheet PMTO 2016). The average number of sessions in the present study was 21.42 (*SD* = 7.90). In 29 % of the PMTO treatments in this study only the foster mother was involved, in 71 % both foster parents attended.

Dutch candidates receive an extensive training (which typically ranges from 18–24 months) to become a certified PMTO therapist with a strong emphasis on program integrity (including training course, structured personal coaching sessions by licensed PMTO supervisors based on recorded sessions of the candidate, national and international assessments). Program integrity is measured with the Fidelity of Implementation Rating System (FIMP; Knutson et al. 2003). The FIMP is based on five categories: PMTO knowledge, structuring, teaching practices, process skills, and overall quality (Knutson et al. 2003). Previous studies show that stronger and more competent therapist adherence to PMTO predicts a stronger improvement of parenting practices and child behavior (Forgatch et al. 2005b; Thijssen 2016). The PMTO candidates’ fidelity to the method is evaluated on a nine-point scale (1–3 needs work, 4–6 acceptable, 7–9 good work; Knutson et al. 2003). To achieve a passing score for PMTO certification, the mean score for two full treatment sessions must be no less than 6.0, with no scores below 4. In the present study, the mean FIMP score of therapists prior to their participation was 7.18 (*SD* = 0.48).

#### Care as Usual

All foster parents received regular support services from the foster care institution. These support services typically included an appointment with a foster care supervisor once every 3–6 weeks. The supervisors were blind for the allocation of families into the control group. If necessary, foster parents from the control group were free to ask for more intensive or specialized support, including every available form of treatment or intervention except PMTO. Foster parents in the intervention group also received care as usual and were free to ask for other help besides PMTO. At posttest, foster parents of both the PMTO and CAU group were asked which (alternative) forms of support or treatment they had received and how often.

### Measures

#### Sociodemographic Variables

Along with the SDQ in the screening phase, the following sociodemographic variables were measured: Age of foster children at baseline, sex (boy/ girl), cultural background (Dutch or non-Dutch), age at entering placement, duration of current placement, number of previous placements, age of foster parents, years of foster parenting experience, family type (one or two parents), placement type (kinship or non-kinship), number of other children in the family and educational background of foster parents (low, middle, or high educated).

#### Experienced Change

We used the Dutch report-form Beste to assess the level of change foster parents experienced after receiving PMTO (Meyer et al. 2004). This parent-report form consists of four items using a five-point scale (1 = change more worse than good, 2 = no change, 3 = some change, 4 = clear and positive change, 5 = do not know). The Beste also includes an item about the experienced length of treatment and whether the parents would advise other parent to follow PMTO treatment. The psychometric qualities of the Beste are acceptable to good (Meyer et al. 2004). The Cronbach’s alpha of the Beste in our sample was (.89 for foster mothers and .78 for foster fathers).

#### Parenting Stress

The Dutch revised version of the Parenting Stress Index (PSI) was used to assess parental experiences of stress and competence in the parenting situation (PSI-R; Abidin 1983; translated revised version by De Brock et al. 1992, 2009, NOSI-R). This parent-report inventory consists of 78 items using a 4-point scale (1 = strongly agree; 4 = strongly disagree) and is divided into 13 subscales (see De Brock et al. 2009), referring to two main domains of parenting stress experience. The “parent domain” (*Parent Stress;* e.g., being a foster parent of this child is more though than I thought it would be, it is difficult to understand what my foster child needs from me; because of being a foster parent, I cannot do other things I would like to do) refers to perceived stress regarding family factors.. The “child domain” (*Child Stress*; e.g., my foster child demands more than my other children, I don’t feel my foster child appreciate my good intentions, a lot of things are upsetting my foster child) refers to stress evoked by their child’s behavior and emotions. Finally, a *Total Stress* score of parenting stress (*Parent Stress* + *Child Stress*) can be calculated. The psychometric qualities of the Dutch version of the PSI-R are acceptable to good (De Brock et al. 1992, 2009). In our sample, the Cronbach’s alphas varied (on baseline and posttest and for foster mothers and fathers) from .67 and .91 for the different subscales. The Cronbach’s alphas of the *Parent*, *Child* and the *Total Stress* score varied from .93 and .97.

#### Parenting Behavior

Parenting behavior was assessed with the Parenting Behavior Questionnaire (PBQ, Wissink et al. 2006). The PBQ comprises 30 items on a 5-point rating scale (1 = never; 5 = very often), divided into six subscales (5 items each), referring to three main dimensions of parental behavior: *warmth* and *responsiveness* (dimension parental support; e.g., how often you compliment your child?), *explaining* and *autonomy granting* (dimension authoritative control; e.g. how often you encourage your child to decide something on its own?) and *strictness* and *discipline* (dimension restrictive control; e.g., how often you need to set strict rules?). In line with previous Dutch studies (De Vries et al. 2016; Kaizer 2009), the Cronbach’s alphas in our sample varied from .59 to .83 for the six different subscales (on baseline and posttest, and for foster mothers and fathers).

#### Child Behavior Problems

Child behavior problems were measured with the Dutch version of the Child Behavior Checklist (CBCL) and the Teacher Report Form (TRF) completed by foster parents and teachers, respectively ( Achenbach 1991). The CBCL and TRF consists of 113 items (6–18 years version, also used for 4–5-years-old after personal agreement of Achenbach) rated on a 3-point Likert scale. *Externalizing Problems* (CBCL: 35 items, TRF: 32 items, e.g., disobedient at home, destroy his/her own things, can’t sit still) and *Internalizing Problems* (CBCL: 26 items, TRF: 27 items, e.g., too fearful or anxious, feels worthless or inferior, worries), the two broadband syndrome scales, along with the *Total Problems* scale, were used in the present study. The *Total Problems* scale includes all behavioral items on the CBCL/TRF and covers externalizing and internalizing problems, thought problems, attention difficulties, and social problems. The psychometric qualities of the Dutch version of the CBCL and TRF are acceptable to good (Evers et al. 2000). The Cronbach’s alphas in our sample varied (on baseline and posttest and for foster mothers and fathers) for *Externalizing Problems* from .90 to .92, for *Internalizing Problems* from .78 to .86 and for *Total Problems* from .85 and .96. The Cronbach’s alphas of the TRF were respectively .93, .89 and .96 (baseline) and .94, .85 and .99 (posttest). The CBCL and TRF were analyzed as separate outcome measures.

### Data Analyses

To check whether the randomization was successful, we compared demographic background variables and baseline outcome measures between the intervention and control condition using *t*-tests and *χ* ^2^ tests. To examine PMTO intervention effects data were analyzed using multilevel regression analysis in which the measures of one or both (if present) foster parents, as well as the repeated measures of the variables included in the study, were considered as nested within participants (Snijders and Bosker 1999). In multilevel analysis, both dependencies between foster parents and dependencies between measurements are taken into account. An additional advantage of multilevel analysis is that all available data can be used, also including data from incomplete cases, without relying on imputation techniques. In total, 15 models were run, predicting parenting stress (total stress, parent, and child related stress), parenting behavior (warmth, responsiveness, explaining, autonomy granting, strictness, discipline) and child behavior problems (total problems, externalizing and internalizing problems, reported by foster parents and teachers). The multilevel (or mixed) regression models included the main effects of condition (PMTO vs. CAU) and parent gender (fathers vs. mothers) *at baseline*, the main effects of time (posttest vs. baseline) and interaction effects with time. To answer our research questions, we were interested in the interaction effects of time and condition. To explicate, all models included an intercept representing the mean score of the foster mother in the CAU group *at baseline*, regression coefficients representing the difference between the PMTO group and CAU group *at baseline*, the difference between fathers and mothers in the CAU group *at baseline* and the additional difference between fathers and mothers in the PMTO group *at baseline*. Next, the models included the change between posttest and baseline for mothers in the CAU group (time effect), the additional change between posttest and baseline for mothers in the PMTO group (time × condition effect), and the additional change between posttest and baseline for fathers in the PMTO group (time × parent gender × condition effect). Please notice that all regression coefficients represent differences and changes in expected outcome scores as estimated under the multilevel regression models. Also note that parent gender does not represent a moderator, but the dependent outcome variable measured by foster mother or father as nested within a family. All outcome variables were standardized, which allows for interpretation of the β coefficients as Cohen’s *d* effect sizes (ESs), with 0.20, 0.50, and 0.80 indicating small, medium, and large ESs, respectively (Cohen 1992). As a result, ESs can be obtained by adding regression coefficients. All analyses were based on the intention to treat principle and thus performed on the total sample (*N* = 86), using statistical package SPSS 22 (IBM Corporation 2011).

For the exploratory moderator analyses, we included regression coefficients in the multilevel regression model for two and three-way interaction effects of condition, moderator, and time. The moderators we explored were child gender, child age, initial severity of externalizing behavior problems and initial severity of maternal depression of foster mothers. The Benjamini-Hochberg False Discovery Rate correction, was used to correct for chance capitalization (i.e., Type 1 errors; Benjamini and Hochberg 2005).

In order to examine whether the amount of change between baseline and posttest was meaningful, we used the Jacobson and Truax’s (1991) method to calculate the Reliable Change Index (RCI) for the dependent variables of each case. In this method, a pretest score is subtracted from a posttest score and this number is than divided by the standard error of difference (S*diff*) between the two test scores. The S*diff* is derived from the standard error (SE) of measurement using the following formula: S*diff* = √(2(SE)^2^). The SE in turn can be derived using the formula SE = (Sd*√1-*r*). If present, we used the standard deviation and the test-retest reliability of the reference data, and otherwise derived the information from our own sample. For the CBCL we had reference information available for the Sd and *r,* for the PSI-R we had reference information available for the Sd. For the PBQ we had no reference data available, and this outcome measure was therefore excluded from further RCI analyses. If the calculated RCI was greater than 1.96 or smaller than −1.96 (*p* < .05), then the change was large enough to be reliable. Then we calculated the percentages of cases that improved (RCI > 1.96) or deteriorated (RCI < −1.96) at posttest. The variability of improvement or deterioration outcomes was too small to use ordinal logistic multilevel analyses to analyze whether the level of *improvement* was significantly higher for PMTO parents compared to CAU. Therefore we did the next best thing and analyzed foster mothers and foster fathers separately. We analyzed with *χ* ^2^ tests the differences between PMTO and CAU group.

## Results

Table 1 shows the demographic characteristics of our sample. The intervention and control group did not differ significantly on demographic characteristics, except for type of family (*χ* ^2^(1) = 8.44, *p* < .001). There were no single-parent families in the intervention group, vs. seven single-parent families in the control group. Excluding single-parent families from the analyses revealed no other time × intervention effects. No significant baseline differences on the outcome measures as reported by both foster parents and teachers were found between the intervention and control group (see Table 2), indicating that the randomization procedure was accomplished successfully and the participants of both conditions were equal on the investigated outcome measures. We found one baseline difference between completers at posttest assessments and drop-outs: foster parents who dropped out had significantly less years of foster experience (*t* = 2.28, *df* = 80, *p* = .03).

**Table 1:** Baseline demographics

	PMTO (*n* = 46)		CAU (*n* = 40)		
	*M n* (%)	SD	*M n* (%)	SD	*p* ^c^
*Demographics*					
Age foster children (years)	7.85	2.36	7.52	2.30	0.51
Sex (boys)	21 (46 %)		20 (50 %)		0.69
Cultural background (non-Dutch)	18 (39 %)		8 (20 %)		0.05
Age at entering placement	3.46	3.12	3.60	2.83	.83
Duration current placement	4.39	2.88	3.92	2.28	.41
Previous placements (*n*)	0.96	0.79	1.05	1.13	.65
Age foster parents (years)^a^	46.55	6.91	48.82	7.79	.16
Foster parent experience (years)^a^	7.80	6.83	7.23	5.47	.68
Family type (one-parent)	0 (0)		7 (18 %)		<.01
Placement type (non-Kinship)	38 (83 %)		34 (85 %)		.76
Other children in family (*n*)	1.67	1.84	1.33	1.49	.34
Educational background foster parents^b^					.26^d^
Low	2 (4 %)		4 (10 %)		
Middle	7 (15 %)		8 (20 %)		
High	37 (80 %)		28 (70 %)		

^a^ No significance differences between mothers and fathers, therefore the mean age is reported

^b^ No significant differences between mothers and fathers, therefore the highest educational level of both foster parents is reported

^c^ Based on *χ* ^2^ or *F* statistics (depending on measurement level)

^d^ Due to small *n*, the low and middle educational background were taken together

**Table 2:** Means and SD’s for parenting stress, parenting behavior and child behavior problems at baseline and posttest

	PMTO				CAU			
	Baseline (*n* = 46)		Posttest (*n* = 30)		Baseline (*n* = 40)		Posttest (*n* = 33)	
	*M*	SD	*M*	SD	*M*	SD	*M*	SD
*Parental Stress (PSI-R)*								
Total scale	156.45	36.15	141.98	36.43	154.48	40.82	158.3	40.82
Parent domain	66.91	18.56	62.07	16.95	66.00	20.03	70.79	22.54
Child domain	88.74	21.28	79.21	22.65	87.67	20.39	83.92	22.49
*Parenting Behavior (PBQ)*								
Warmth	4.10	0.62	4.10	0.67	4.16	0.63	4.14	0.61
Responsiveness	3.80	0.66	3.89	0.55	3.88	0.57	3.90	0.60
Explaining	4.01	0.56	3.98	0.60	4.12	0.57	4.09	0.50
Autonomy granting	3.18	0.56	3.38	0.59	3.28	0.50	3.51	0.52
Strictness	3.09	0.55	2.78	0.62	3.24	0.57	3.18	0.53
Discipline	2.26	0.58	2.12	0.61	2.21	0.56	2.24	0.53
*Child Behavior (T score CBCL)*								
Total problems	65.64	8.89	60.63	10.62	66.25	7.14	63.00	9.19
Externalizing problems	66.43	9.06	62.10	10.09	67.13	8.09	64.75	9.68
Internalizing problems	58.83	9.36	54.91	10.35	57.67	9.96	53.89	10.92
*Child Behavior (T score TRF)*								
Total problems	59.43	7.76	58.07	9.12	61.08	8.46	62.03	9.40
Externalizing problems	81.19	20.55	77.86	22.11	80.97	19.65	81.59	19.60
Internalizing problems	54.98	10.09	55.32	9.92	55.22	10.47	55.69	10.18

In the CAU group 21 foster parents (63 %) reported at posttest measurement that they received some form of alternative parenting support or psychological treatment for their child in the last 6 months. Three cases concerned fully protocolled interventions comparable to PMTO (i.e., Triple P course, Video Interaction Guidance, Intensive Home Treatment), two cases concerned a few parent consults related to the treatment of the child and the other cases concerned individual child treatment (e.g., play therapy, music therapy, EMDR, psychological counseling, sensory integration therapy). Also 13 foster parents (43 %) in the PMTO group received alternative support or treatment in addition to PMTO (once social work for the foster parents, in the other cases the support or treatment was aimed at the foster child, including homeopathy, sensory therapy, therapy from mental health organization, child-coaching). The number of families in the CAU and PMTO group that received additional support did not differ significantly.

In the PMTO group, 77 % of the foster mothers and 79 % of the foster fathers experienced the treatment as effective. Moreover, 80 % of the foster mothers was satisfied about the length of the treatment and the same percentage would definitely recommend PMTO to other parents. 67 % of the foster fathers was satisfied about the length of the treatment and 78 % would definitely recommend PMTO to other parents.

### Main Effects of PMTO

Table 3 shows the results of the multilevel analyses. In this section, we report the results of the first outcome measure PSI-R *Total Stress* scale row by row. For the other outcome measures we only mention the relevant results in relation to the research questions. We note that the *SE* information in Table 3 gives an idea of the confidence intervals of the beta weights (lower/upper bounds of a 95 % confidence interval are approximately equal to the point estimate ± 1.96 × SE).

**Table 3:** Intervention effects for PMTO vs. care as usual for parental stress (PSI-R), parenting behavior (PBQ) and child behavior (CBCL)

	PSI-R Total			PSI-R Parent			PSI-R Child		
	*β*	*SE*	*p*	*β*	*SE*	*p*	*β*	*SE*	*p*
Intercept^a^	0.15	0.15	.30	0.16	0.16	.32	0.12	0.14	.39
Differences at baseline									
Condition^b^	0.01	0.20	.95	−0.11	0.21	.62	0.11	0.19	.58
Parent gender^c^	−0.33	0.14	.02	−0.39	0.15	.01	−0.20	0.13	.15
Parent gender × condition^d^	0.09	0.18	.64	0.29	0.20	.14	−0.11	0.18	.53
Differences between posttest and baseline									
Time^e^	0.11	0.09	.24	0.24	0.10	.02	−0.02	0.10	.86
Time × condition^f^	−0.48	0.14	.00	−0.54	0.14	.00	−0.34	0.14	.02
Time × parent gender^g^	−0.18	0.16	.28	−0.22	0.16	.17	−0.08	0.17	.63
Time × parent gender × condition^h^	0.25	0.22	.26	0.40	0.21	.06	0.05	0.23	.83

	PBQ warmth			PBQ responsiveness			PBQ autonomy granting		
	*β*	*SE*	*p*	*β*	*SE*	*p*	*β*	*SE*	*p*
Intercept^a^	0.35	0.11	.00	0.14	0.14	.32	−0.02	0.15	.87
Differences at baseline									
Condition^b^	0.10	0.14	.51	0.20	0.19	.29	0.05	0.20	.81
Parent gender^c^	−0.68	0.17	.00	−0.24	0.18	.19	−0.05	0.18	.77
Parent gender × condition^d^	−0.39	0.22	.08	−0.76	0.24	.00	−0.55	0.24	.02
Differences between posttest and baseline									
Time^e^	−0.22	0.09	.01	0.08	0.14	.56	0.44	0.15	.01
Time × condition^f^	0.35	0.13	.01	0.09	0.20	.64	−0.17	0.22	.42
Time × parent gender^g^	0.36	0.15	.02	−0.27	0.22	.23	−0.22	0.24	.38
Time × parent gender × condition^h^	−0.41	0.20	.04	0.29	0.29	.33	0.35	0.32	.29

	PBQ Explaining			PBQ Strictness			PBQ Discipline		
	*β*	*SE*	*P*	*β*	*SE*	*p*	*β*	*SE*	*p*
Intercept^a^	0.28	0.14	.05	0.42	0.15	.01	0.00	0.15	.99
Differences at baseline									
Condition^b^	0.03	0.19	.87	−0.24	0.21	.25	0.02	0.20	.91
Parent gender^c^	−0.42	0.19	.03	−0.40	0.16	.01	−0.05	0.16	.78
Parent gender × condition^d^	−0.43	0.26	.10	0.05	0.21	.83	0.18	0.21	.38
Differences between posttest and baseline									
Time^e^	−0.10	0.11	.37	−0.24	0.13	.08	−0.09	0.13	.51
Time × Condition^f^	0.15	0.16	.35	−0.30	0.19	.12	−0.15	0.19	.43
Time × Parent gender^g^	−0.03	0.20	.87	0.01	0.20	.96	0.14	0.18	.42
Time × Parent gender × condition^h^	−0.05	0.27	.85	0.25	0.27	.36	−0.08	0.23	.73

	CBCL Total			CBCL Externalizing			CBCL Internalizing		
	*β*	*SE*	*p*	*β*	*SE*	*p*	*β*	*SE*	*p*
Intercept^a^	0.21	0.15	.16	0.15	0.16	.36	0.19	0.17	.26
Differences at baseline									
Condition^b^	0.12	0.20	.56	0.12	0.21	.56	0.03	0.23	.89
Parent gender^c^	−0.18	0.14	.21	−0.07	0.12	.54	−0.28	0.17	.10
Parent gender × condition^d^	−0.16	0.18	.39	−0.28	0.15	.07	0.12	0.22	.59
Differences between posttest and baseline									
Time^e^	−0.26	0.12	.03	−0.16	0.12	.19	−0.35	0.14	.02
Time × Condition^f^	−0.16	0.17	.33	−0.33	0.17	.06	0.14	0.20	.50
Time × Parent gender^g^	−0.08	0.15	.59	−0.14	0.15	.36	0.15	0.18	.40
Time × Parent gender × condition^h^	0.12	0.21	.56	0.37	0.20	.07	−0.34	0.25	.17

	TRF Total			TRF Externalizing			TRF Internalizing		
	*β*	*SE*	*p*	*β*	*SE*	*p*	*β*	*SE*	*p*
Intercept	0.12	0.15	.44	0.16	0.15	.30	0.01	0.17	.97
Differences at baseline									
Condition	−0.26	0.21	.22	−0.36	0.21	.09	−0.05	0.23	.84
Differences between posttest and baseline									
Time	0.16	0.19	.41	0.13	0.17	.47	0.06	0.16	.72
Time × Condition	−0.13	0.27	.63	−0.04	0.25	.88	−0.03	0.23	.89

*Note*: All outcome variables have been standardized so ESs can be obtained by adding regression coefficients (*β*) and interpreted with 0.20, 0.50, and 0.80 indicating small, medium, and large effect sizes (Cohen 1992)

^a^ Mothers in CAU at baseline

^b^ Difference between PMTO and CAU groups, for mothers, at baseline

^c^ Difference between fathers and mothers in CAU group, at baseline

^d^ Additional difference for fathers (vs. mothers) in PMTO group (vs. CAU group)

^e^ Change (posttest vs. baseline) in mothers in CAU group

^f^ Additional change in mothers in PMTO group (vs. CAU group)

^g^ Additional change in fathers (vs. mothers) in CAU group

^h^ Additional change in fathers (vs. mothers) in PMTO group (vs. CAU group)

#### Effect of PMTO on Parenting Stress (PSI-R)

Table 3 first shows the main effects for condition, time, and parent gender on PSI-R Total Stress scale. At baseline, there were no significant differences between the CAU and PMTO condition (*β* = 0.01, *p* = .95). There was a baseline difference between foster mothers and fathers. Foster fathers in CAU reported significantly lower total stress levels than foster mothers in CAU (*β* = −0.33, *p* = .02). There was no additional change in the PMTO group (*β* = 0.09, *p* = .64), indicating that there was a same baseline difference for foster mothers and foster fathers in the PMTO group. At posttest, there was no significant change for foster mothers in the CAU group (*β* = 0.11, *p* = .24). The additional change for foster mothers in the PMTO group was significant (*β* = −0.48, *p* < .00). That is, compared to foster mothers in CAU, foster mothers who received PMTO significantly reported lower stress levels (time × intervention effect for foster mothers = −0.48, medium effect). The additional change for foster fathers in the CAU group was not significant (*β* = −0.18, *p* = .28) and neither for foster fathers in the PMTO group (*β* = 0.25, *p* = .26). The time × intervention effect for foster fathers can be computed as −0.48 + 0.25 = −0.23 (small effect). Thus, the effect of PMTO was stronger for foster mothers than for foster fathers, but this difference was not significant.

Overall, compared to CAU, PMTO resulted in a significantly stronger decrease of total parenting stress (ES foster mothers: −0.48; ES foster fathers: −0.48 + 0.25 = −0.23) and child related parenting stress (ES foster mothers: −0.34; ES foster fathers: −0.34 + 0.05 = −0.29). In addition, PMTO significantly reduced parent-related parenting stress (ES foster mothers: −0.54; ES foster fathers: −0.54 + 0.40 = −0.14), while this stress significantly increased in the CAU group (ES mothers: 0.24; ES foster fathers: 0.24 + −0.22 = 0.02). The differences between foster mothers and foster fathers for PMTO effects were not significant.

#### Effect of PMTO on Parenting Behavior

There were no significant effects of PMTO, compared to CAU, on the parenting behaviors of responsiveness, autonomy granting, explaining, strictness and discipline. However, there was one significant effect of PMTO, compared to CAU, on parental warmth of foster mothers (ES: 0.35), but not for foster fathers (ES: 0.35 + −0.41 = −0.06). The warmth of the foster mothers in the PMTO group (ES: −0.22 + 0.35 = 0.13) and the foster fathers in the CAU group (ES: −0.22 + 0.36 = 0.14) slightly increased, while the warmth of foster mothers in the CAU group decreased (ES: −0.22) and warmth of the foster fathers in the PMTO group remained stable (ES: 0.35 + −0.41 = −0.06). These effect sizes are significantly different from each other, but were all rather small (see Table 3).

#### Effect of PMTO on Child Behavior (CBCL and TRF)

Compared to CAU, there was no significant effect of PMTO on total problems, externalizing problems and internalizing problems as reported by foster mothers (ESs were −0.16, −0.33 and 0.14, respectively), foster fathers (ESs were −0.16 + .012 = −0.04, −0.33 + 0.37 = 0.04, and 0.14 + −0.34 = −0.20, respectively) and teachers (ESs were −0.13, −0.04, and −0.03, respectively). Although over time parent-reported total problems in the PMTO group significantly decreased (ES foster mothers: −0.26 + −0.22 = −0.42; ES foster fathers: −0.26 + −0.22 + 0.12 = −0.30), this decrease was not significantly different from the decrease in the CAU group (ES foster mothers: −0.26; ES foster fathers: −0.26 +  −0.08 = −0.34). Similarly, although over time there was a significant decrease of parent reported internalizing problems in the PMTO group (ES foster mothers: −0.35 + 0.14 = −0.21; ES foster fathers: −0.35 + 0.14 + −0.34 = −0.55), this decrease was not significantly different from the decrease in the CAU group (ES foster mothers: −0.35; ES foster fathers: −0.35 + 0.15 = −0.20). No significant decrease over time was observed in both conditions for parent-reported externalizing problems and all teacher-reported problem scales. Furthermore, no significant differences between foster mothers and foster fathers were observed in PMTO effects.

#### Moderator Effects

We found no significant moderating effects of child gender, child age, initial severity of externalizing behavior problems, and initial severity of maternal depression of foster mothers on parenting stress, parenting behaviors, and child behavior problems.

#### Clinical Significance

Table 4 shows information about the number of foster mothers and fathers who showed reliable improvement or deterioration regarding their parenting stress and or regarding the reported problem behavior of their foster child. Fisher exact tests revealed no differences between the PMTO and CAU group.

**Table 4:** Reliable change (Improvement or Deterioration) on parenting stress parent-reported child behavior problems

		PMTO			CAU			
		Clinical at baseline	I	D	Clinical at baseline	I	D	*p*
		*N* (%)	*N* (% of total)	*N* (% of total)	*N* (%)	*N* (% of total)	*N* (% of total)	
PSI-R Total scale	fostermother	30 (65)	4 (13)	1 (3)	22 (58)	1 (3)	2 (7)	.29
	fosterfather	21 (53)	2 (7)	1 (4)	14 (47)	2 (10)	0 (0)	.60
PSI-R Parent domain	fostermother	12 (26)	4 (13)	1 (3)	11 (29)	1 (3)	3 (10)	.17
	fosterfather	13 (33)	4 (15)	3 (11)	8 (27)	2 (10)	1 (5)	.67
PSI-R Child domain	fostermother	36 (78)	5 (17)	0 (0)	28 (74)	2 (7)	3 (10)	.08
	fosterfather	24 (60)	3 (11)	1 (4)	19 (63)	3 (14)	0 (0)	.57
CBCL total problems	fostermother	32 (70)	13 (43)	2 (7)	30 (79)	9 (29)	3 (10)	.39
	fosterfather	23 (58)	10 (37)	2 (7)	20 (67)	10 (48)	3 (14)	.54
CBCL externalizing problems	fostermother	31 (67)	14 (47)	3 (10)	25 (66)	7 (23)	5 (16)	.16
	fosterfather	20 (50)	9 (33)	4 (15)	21 (70)	6 (29)	2 (10)	.63
CBCL internalizing problems	fostermother	17 (37)	2 (7)	2 (7)	16 (42)	5 (16)	2 (7)	.47
	fosterfather	12 (30)	7 (26)	1 (4)	7 (23)	3 (14)	1 (5)	.50

*Note*: RCI classification based on Jacobson and Truax (1991)

*p* represents Fisher’s exact test

*I* Improved, *D* Deterioration

#### Effects of Additional Care

Additional analyses were conducted to gain insight in the possible influence of additional care on parenting stress, parenting behavior, and child problem behavior effects. The exclusion of the CAU families who received other forms of evidence-based parenting interventions, revealed no other results. The same applied when the families were excluded if the foster child received child treatment. For the PMTO participants, we found no differences in effect for families who received other support (in addition to PMTO) of families who only received PMTO.

## Discussion

This RCT in a Dutch real-world foster care setting tested the effectiveness of PMTO to reduce foster parents’ stress, improve foster parents’ parenting behavior and to reduce child behavior problems. The study targeted families of foster children (aged 4–12) with severe behavioral problems and placed within long-term foster care arrangements. PMTO did significantly reduce general levels of parenting stress, specific parent-related stress (e.g., feeling depressed or incompetent) and child related stress (e.g., adaptability, demandingness of the child). The clinical significance of this effect was, however, limited. The effect of PMTO on foster mothers’ stress levels (medium effect sizes) was stronger than for foster fathers (small effect), although this difference was not significant. There was a small positive effect of PMTO on foster mothers’ (but not fathers’) ability to maintain parental warmth, but no effects on other self-reported parenting behaviors. We found, however, that child behavior problems were equally reduced in the PMTO and in the care as usual control condition. Additional analyses showed that child gender, age, initial levels of child behavior problems, and parenting stress did not appear to play a role in the effects of PMTO.

The effect of PMTO on reduced parenting stress, overall and specific child and parent-related stress, is promising and justifies that the role of parenting stress into the PMTO model should be further studied. To our knowledge, no previous research investigated the effect of PMTO on parenting stress and, more broadly, parenting stress has been investigated only scarcely as an outcome measure in other intervention studies in foster care. The limited clinical impact of PMTO on reduced parenting stress at an individual level, however, makes clear that we need to be careful in our conclusion about the effect of PMTO on parenting stress. What our findings indicate is that PMTO may help foster parents to perceive the functioning of their foster child as less stressful. We may assume that the high intensity of PMTO provided by highly qualified professionals familiar to the difficulties of foster children, helps foster parents in such a way that they more easily accept their child’s behavior and formulate more appropriate expectations. This may support foster parents to keep going (especially in long-term foster placements) when child rearing processes are challenging. This is an encouraging finding, because it is well-known that foster parents who experience elevated levels of parenting stress are at increased risk of placement breakdown (Vanderfaeillie et al. 2012; Van Rooij et al. 2015). Furthermore, enhanced psychological functioning of foster parents appears to be essential to foster carer retention (Turner et al. 2009). Follow-up studies to find out whether reduced stress levels indeed result in enhanced placement stability are needed. Moreover, it is important to examine whether PMTO specific factors or intervention non-specific processes are of key importance to produce this effect (Assay and Lambert 1999; Duncan et al. 2010).

While maternal warmth in the care as usual condition decreased, PMTO especially seems to help foster mothers to maintain their level of parental warmth. This is positive since emotional involvement of parents is supposed to mediate improved functioning of children (Patterson 2005). Notwithstanding this small effect, the lack of a main effect on other self-reported parenting behavior was not expected for three reasons. First, earlier PMTO studies have shown main effects on parenting (e.g., Forgatch et al. 2005a; Ogden and Hagen 2008). Second, parenting behaviors are the presumed mechanism of change in child adjustment within the SIL program theory (Patterson 2005). The child behavior problems did reduce and we thus expected that PMTO would have affected parenting behavior. Third, a body of other research shows that positive parenting improves when feelings of parental competence and wellbeing increase (Jones and Prinz 2005). In this study, PMTO helped to reduce parenting stress, however, without changing parenting behavior. So the question is—what makes it so hard to change foster parenting behavior (see also Bywater et al. 2010; MacDonald and Turner 2005)? It may be that foster carers differ from other parents in their parenting behavior. First, they are initially selected with the assumption to have sufficient parenting skills to manage the range of disruptive behaviors encountered in (or by) children with such troubled backgrounds as those typically placed in foster care (Lindsey 2001). Second, the process of coercive cycles in foster parent-child dyads may develop differently from biological parent-child dyads, because the origins of the child’s behavioral problems often are from neglectful and/or abusive parenting from former caregivers, but not from the current foster parent and foster parents and children have spent less time together (see also Leve et al. 2012; Timmer et al. 2006). Further research on differences in treatment goals between PMTO for foster parents and biological parents would help to understand (1) whether they indeed have different parenting needs, and (2) what this implies for interventions aimed at improving foster parenting behavior.

Given the few changes in parenting behaviors, the absence of an effect of PMTO, compared to a care as usual condition, on child behavior problems is not surprising. We expected, however, that the individualized and intensive format of PMTO would have reached clear effects that other, lighter interventions were unable to achieve (Dorsey et al. 2008; Turner et al. 2009). Although the majority of the foster parents who received PMTO were very satisfied about the perceived effect and our findings indeed do not refute the potential efficacy of PMTO, they do question the added value of PMTO to care as usual to reduce behavioral problems in this foster care population. The substantial reductions of child behavior problems in the control group suggest relatively high quality of standard care as usual for foster parents in The Netherlands. Similar patterns have been observed in other European countries (e.g., Sweden: Sundell et al. 2008). In our sample, care as usual meant that foster parents at least attended regular assisting services (once in the 3–6 weeks). A majority of the families also used forms of additional parenting or child support. Though less intensive compared to PMTO, the use of multidimensional support may have affected the outcomes on child behavior measures comparable to wrap around programs as TFC tend to have (Kinsey and Schlosser 2013). Another, statistical, explanation for the absence of an intervention effect on child behavior problems, is that a regression to the mean (RTM) effect might have occurred (Barnett et al. 2005). This means that when selecting a non-random sample (in our study a sample with elevated child behavior problems), there always is a posttest effect toward the population mean, irrespective to what happened (or not) between pretest and posttest. Though we tried to reduce this RTM effect as much as possible by the use of a stringent study design (RCT) with multiple outcome measures, it cannot be excluded completely.

Why did PMTO affect parenting stress when it did not improve, more than care as usual, child functioning or parenting practices? One possible explanation is that parenting stress of foster parents may be caused by more or other factors than just the difficulties to manage disruptive child behaviors. For example, complicated contact with biological parents or a lack of say in foster child’s future are also challenging factors and reasons for foster parents to quit fostering (Rhodes et al. 2001). Although perhaps unintended and in an indirect way, the intensive PMTO treatment might support foster parents to better cope with these additional problems, which increases their personal wellbeing while in fact their parenting behavior or child behavior does not necessarily change. This points to the necessity to further investigate how principles of multidimensional and intensive TFC programs, can be effectively implemented in Dutch long-term foster care. We can also hypothesize that there will be a “sleeper effect”, indicating that changes in reported parenting and child behavior become more pronounced at later follow-up (e.g., Barlow et al. 2007; Whittingham et al. 2009). A meta-analysis on early developmental prevention programs (e.g., family support services) delivered to at-risk populations, even demonstrates positive effects on individual and family well-being into adolescence (Manning et al. 2010). Indeed, a growing number of studies show that senses of parental competence and wellbeing, including reduced parenting stress, mediate the effect of parenting interventions on improved parenting practices and child problem behavior (e.g., Deković et al. 2010; Hermanns et al. 2013). This suggests that effects of PMTO on reduced parental stress may in the future lead to positive changes in parenting behavior and possibly child functioning. However, this explanation may not be the most convincing, given that a recent meta-analysis on immediate and follow-up effects of parenting interventions on reduced disruptive child behavior found no evidence for sleeper effects of parenting interventions (Van Aar et al. in preparation).

We found no moderation of PMTO effects by child gender, age, initial levels of child behavior problems or parenting stress. The small sample size may have limited the power to detect potential moderator effects. Though another recent large-scaled RCT on the effectiveness of IY in the Netherlands also found no evidence for moderation of IY effects on a variety of previously suggested moderators (e.g., child gender, initial severity of behavioral problems; Weeland et al. in press). Although not significant (except for maternal warmth), the differences in effect sizes tend to indicate that that foster mothers benefited more than foster fathers from PMTO. This is in line with meta-analytic findings that fathers tend to report fewer desirable gains from parenting interventions than mothers (Lundahl et al. 2008). Foster fathers in our sample also reported lower levels of parenting stress and child behavior problems at baseline than foster mothers, and thus had less room for improvement. Moreover, more foster mothers than foster fathers were involved in PMTO treatment. Their goals may have therefore been most dominant in the PMTO sessions. Since previous research has shown that the involvement of fathers contributes to the overall effects, also for mothers, their attendance still needs to be encouraged (Lundahl et al. 2008).

Some study limitations must be taken into account. Maybe because of selecting a high risk sample, there was a relatively high drop-out rate at posttest (PMTO: *n* = 16 (35 %), CAU: *n* = 7 (18 %)). In the intervention group, foster parents dropped out before starting PMTO. Moreover, all foster parents who started with the intervention also finished PMTO and posttest assessments. On the one hand, the five families who dropped out because their foster child moved out of the family, underpins the considerable risk for placement disruptions in this sample. On the other hand, the seven families who rejected PMTO because they felt no need for help, indicates that at least for a part of our sample, the presence of child behavior problems not necessarily implies a need for this kind of support. In spite of dropout, statistical power of the multilevel analysis was still sufficient. For example, for parenting stress, we observed 0.75 correlations between measurements and 0.60 correlations between parents, yielding 87 % power to detect medium sized interaction effects at a 5 % level of significance. Another procedural limitation is that due to the two screening phases (with the SDQ and PDR) previous to allocation to the RCT, foster parents were aware of the research focus on child behavior problems. This might have influenced their posttest scores. A final limitation is that the outcome measures are based on self-reports. Additional observational data might have revealed other results.

Despite these limitations, the present study has several specific strengths also. First, this is one of the first studies outside the United States to test the effectiveness of parent training in a foster care setting using a stringent RCT design. Contrary to efficacy trials conducted in controlled research contexts, the importance of an effectiveness trial such as this study, is to test whether an intervention works in a real-life setting (Hoagwood et al. 1995; Weisz 2014). These trials are scarce in foster care. The validity of this study is strengthened by a successful randomization of a complex and high risk target group. Other strengths of the present study are its multi-informant design (both foster parents when applicable and teacher) and the focus of PMTO effects on parenting stress, parent-reported child behavior problems and parenting practices following a theoretical analysis of foster-family placements breakdown.

To conclude, this study shows that PMTO within long-term foster care did reduce foster parenting stress and did help foster mothers to maintain their parenting warmth. However, PMTO had no convincing, systematic effect on improved parenting behavior and had no effects above and beyond the effects of care as usual on reduced foster child behavior problems. The effect on reduced parenting stress is promising; if foster parents are less stressed, they generally feel more competent in their parenting and perhaps experience that they can handle their foster child’s behavior problems more easily in the long term and in doing so reduce problematic behavior of foster children. In this way, lower levels of stress may be important for increasing placement stability, which in turn is important for improving foster children’s outcomes. Long-term follow-up studies are however necessary to corroborate this line of reasoning.

The authors declare that they have no conflict of interest. The study received ethical approval from the Ethical Committee of the Research Institute of Child Development and Education of the University of Amsterdam. This article does not contain any studies with animals performed by any of the authors. Informed consent was obtained from all individual participants included in the study.
